# Expression of Neighbor of Punc E11 (NOPE) in early stage esophageal adenocarcinoma is associated with reduced survival

**DOI:** 10.1038/s41598-022-07580-y

**Published:** 2022-03-04

**Authors:** Fabian Kütting, Florian Gebauer, Susanne Zweerink, Laurenz Krämer, Christoph Schramm, Alexander Quaas, Christiane Bruns, Tobias Goeser, Dirk Nierhoff

**Affiliations:** 1 Department of Gastroenterology and Hepatology, University Hospital of Cologne, University of Cologne, Kerpener Str. 62, 50937 Cologne, Germany; 2 Department of General, Visceral, Cancer and Transplantation Surgery, University Hospital Cologne, University of Cologne, Cologne, Germany; 3 Institute of Pathology, University Hospital Cologne, University of Cologne, Cologne, Germany

**Keywords:** Liver cancer, Oesophageal cancer

## Abstract

Current recommendations suggest neoadjuvant treatment in node-positive esophageal cancer or tumors staged T3 and upwards but some T2 N0 patients might benefit from neoadjuvant therapy. It is of clinical relevance to identify this subgroup. Loss of epithelial apicobasal polarity is a key factor in the development of invasive capabilities of carcinoma. The oncofetal stem/progenitor cell marker NOPE is expressed in adult depolarized murine hepatocytes and in murine/human hepatocellular carcinoma. We analyzed NOPE expression in 363 patients with esophageal adenocarcinoma using an RNA Scope Assay on a tissue microarray and correlated results with clinical data. Median follow-up was 57.7 months with a 5-year survival rate of 26.6%. NOPE was detectable in 32 patients (8.8%). In pT1/2 stages, NOPE expression was associated with a significantly reduced median OS of 6.3 months (95% CI 1.2–19.4 months), the median OS is not reached in the NOPE-negative group (calculated mean OS 117.1 months) (*P* = 0.012). In advanced tumor stages, a NOPE dependent survival difference was not detected. This is the first report of NOPE expression demonstrating a prognostic value in esophageal cancer. Early stage, NOPE positive patients are at a high risk of tumor progression and may benefit from neoadjuvant treatment analogous to advanced stage cancer.

## Introduction

Esophageal carcinoma (including squamous cell carcinoma and adenocarcinoma) is the 8th most commonly diagnosed cancerworldwide^1^. The incidence of esophageal adenocarcinomas (EAC) has increased over recent decades^2,3^, a phenomenon observed mainly in the Western world. In the United States adenocarcinoma incidence increased sevenfold from only 3.6 per million (1973) to 25.6 per million in 2006. This progression has since slowed down. The current combined incidence for EAC and squamous cell carcinoma esophageal cancer in the United States lies at 58.3 per million (58.4% EAC), resulting in a current EAC incidence of 3.4 per million^4,5^.

The overall ongoing increase in sincidence likely lies in higher incidence of known risk factors such as obesity^6^. Other risk factors include smoking, a history of gastroesophageal reflux, and low fruit and vegetable consumption^7^.

Adenocarcinomas of the esophagus develop from Barrett mucosa caused by chronic reflux disease^8–10^. An accumulation of mutations, copy-number variations, in part due to chromothripsis ultimately leads to an environment of genetic instability that induces an adenocarcinoma^11^.

Despite improvements in perioperative treatment regimens, overall survival of patients with esophageal carcinoma remains low. The 5-year relative survival rate is poor with 21% (adenocarcinoma: 20.2%; squamous cell carcinoma: 22.8%)^12,13^. Due to a lower rate of lymph node metastasis and occult distant metastases, the T2 category has a prognostic advantage over T3/4 tumors and the expected benefit derived from neoadjuvant therapy is probably lower^14,15^. Therefore, current clinical recommendations suggest neoadjuvant treatment in tumors staged from N+ or T3 Tumors upwards^16^. Regardless, some T2 N0 patients appear to be biologically understaged in terms of their ultimate postoperative survival. It is of clinical relevance to identify those patients who are in a macroscopically early stage and to identify histological markers apart from known markers such as integrin alphaV (ITGAV), a transmembrane glycoprotein responsible for cell-to-matrix binding^17^ and Indoleamine 2,3-dioxygenase (IDO), an interferon-inducible immune checkpoint^18^, to distinguish a subgroup with a poor prognosis and imminent tumor progression beyond the primary site.

In esophageal cancer, the loss of epithelial apicobasal polarity is a key factor in the induction of oncogenesis, tumor progression, and the development of the tumors´ invasive capabilities^19^. A similar effect was shown in liver cancer by Wan et al., who described how the loss of epithelial cell polarity can play a vital role in the development and progression of liver cancer^20^. The oncofetal stem/progenitor cell marker Neighbor of Punc E11 (NOPE) is regularly expressed in adult depolarized murine hepatocytes induced by acute or chronic cholestatic liver injury^21^ as well as in murine and human hepatocellular carcinoma (HCC)^22,23^. In human HCC, NOPE has a superior sensitivity in non-cirrhotic livers compared to the standard marker AFP and has been shown to be complementary to GPC-3^23^. HCCs are usually caused by chronic inflammation (viral or non-/alcoholic hepatitis e.g.) or consecutive cirrhosis and show high molecular diversity and genomic instability. Similarly, EACs develop at the base of chronic inflammation due to reflux esophagitis and are characterized by "genomic chaos"^24^. Despite the differences between the two entities, there are relevant overlaps, which led us to analyze EAC for its distribution and relevance of NOPE expression. Therefore, we decided to investigate the relevance of NOPE expression in 363 patients with EAC analyzing mRNA-expression of NOPE using RNA-scope technology in a conserved tissue context and correlate the expression pattern with clinical (survival) data.

## Material and methods

### Patients and tumor samples

Formalin-fixed and paraffin embedded material of patients with EAC that underwent primary surgical resection or resection after neoadjuvant therapy between 1999 and 2015 at the Department of General, Visceral and Cancer Surgery, University Hospital Cologne, Germany was analyzed. All procedures performed in studies involving human participants were in accordance with the ethical standards of the 1964 Helsinki declaration and its later amendments or comparable ethical standards. This study was approved by the University of Cologne Ethics Committee (BioMasota Project, reference no. 13-091) and written informed consent was obtained from all patients. Standard surgical procedures were laparotomic or laparoscopic gastrolysis and right transthoracic en bloc esophagectomy with intrathoracic esophagogastrostomy including two-field lymphadenectomy of mediastinal and abdominal lymph nodes or transhiatal extended distal esophagectomy with transabdominal intrathoracic or cervical anastomosis as described previously^25^.

All Patients with advanced esophageal cancer (cT3 minimum) or detection of lymph node metastasis were treated with preoperative chemoradiation (5-Fluouracil, cisplatin, 40 Gy) or chemotherapy. Follow-up data were available for all patients. Response to neoadjuvant chemo- or radiochemotherapy was defined as minor residual tumor of ≥ 10% found in histopathological analysis^26^.

### Tissue microarray

In accordance with the suggestions of the international immunooncology working group for assessing TILs on solid tumors, we constructed a tissue-micro array (TMA)^27–30^. Construction of the TMA and immunohistochemical staining procedures were performed as described previously^27,28^. Tissue cylinders with a diameter of 1.2 mm each were punched from selected tumor tissue blocks using a self-constructed semi-automated precision instrument and embedded in empty recipient paraffin blocks.

### RNA-scope-assay

We performed an RNAscope assay according to the manufacturer’s instructions^31^. Paraffin-embedded TMA blocks were cut in 5 μm sections, pretreated according to extended protocol (30 min for pretreatment 2 and 3), digested and hybridized at 40 °C in the HybEZ oven with human NOPE mRNA probe provided by Advanced Cell Diagnostics Europe. Incubation time with Hematoxylin was 10 s. Target expression was compared to both negative (dapB) and positive (PPIB) controls. Scoring of signals was carried out as recommend by the manufacturer with no staining or less than one molecule per 10 cells = score 0, positivity was defined as a score > 0.

### Correlation of NOPE with other immunohistochemical markers

NOPE expression was analyzed for correlation with immunohistochemical markers including integrin alphaV expression (ITGAV) and Indoleamine 2,3-dioxygenase (IDO) expression on tumor infiltrating lymphocytes (TILs) and tumor cells. Immunohistochemistry (IHC) was performed on TMA slides using the monoclonal rabbit anti—ITGAV antibody (ab150361; dilution 1:300; Abcam, UK) and the monoclonal rabbit IgG anti—IDO antibody (D5J4E; dilution 1:400; Cell Signaling Technology, USA). Both staining procedures are described elsewhere in detail^17,18^.

### Statistical analysis

Clinical data were collected prospectively according to a standardized protocol. SPSS Statistics for Mac (Version 21, SPSS) was used for statistical analysis. Interdependence between staining and clinical data were calculated using the chi-squared and Fisher’s exact tests, and displayed by cross-tables. Survival curves were plotted using the Kaplan–Meier method and analyzed using the log-rank test. Univariate and multivariate analyses were performed for prognostic factors of overall survival using the Cox regression model. All tests were two-sided. *P* values < 0.05 were considered statistically significant.

## Results

Out of 481 patients with EAC on the TMA, 363 patients were analyzable for NOPE (75.5%) and were subsequently considered for analysis. Reasons for non-informative cases included lack of tissue samples or absence of unequivocal cancer tissue in the TMA spot. The median follow-up for the entire cohort was 57.7 months with a calculated 5-year survival rate of 26.6%. Patient characteristics are given in Table 1.

**Table 1:** Baseline characteristics.

	Number	Percent	NOPE neg	Percent	NOPE pos	Percent	*P* value
			Number		Number		
**Sex**							
Female	37	10.2	30	81.1	7	18.9	0.032
Male	326	89.8	301	92.3	25	7.7	
**Age group**							
< 65	182	53.2	164	90,1	18	9.9	ns
> 65	160	46.8	148	92.5	12	7.5	
**Tumor stage**							
pT1	40	11	40	100	0	0	ns
pT2	30	8.3	26	86.7	4	13.3	
pT3	282	77.7	256	90.8	26	9.2	
pT4	11	3	9	81.8	2	18.2	
Total	363	100	331	91.2	32	8.8	
**Lymph node metastasis**							
pN0	133	36.6	123	92.5	10	7.5	ns
pN +	230	63.4	208	90.4	22	9.6	

**Figure 1:**
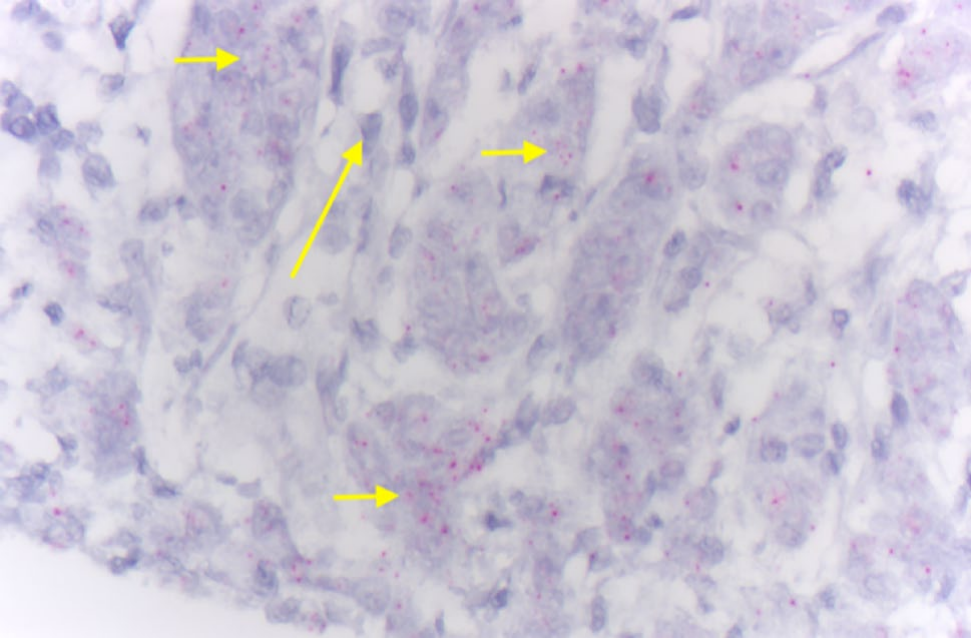
Adenocarcinoma of the esophagus shows homogeneous mRNA in situ positivity (small red dots) in the carcinoma cells (short arrow), surrounding stromal cells such as fibroblasts and endothelia are NOPE mRNA negative (long arrow).

**Figure 2:**
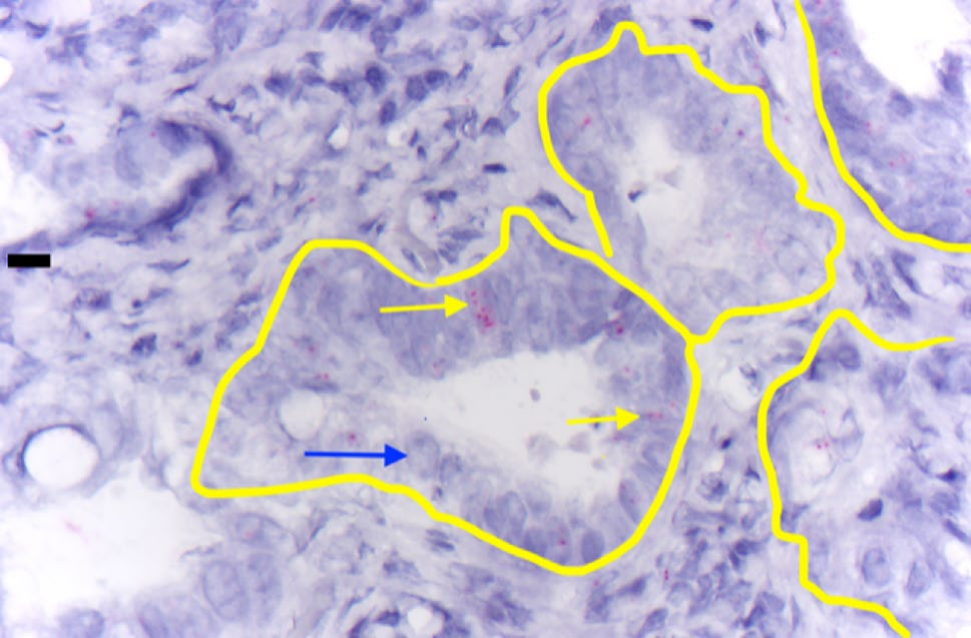
Tubular-intestinal differentiated adenocarcinoma of the esophagus is heterogeneously positive for NOPE mRNA (multiple wide lumen carcinoma glands are outlined in yellow). The positive carcinoma cells are exemplarily marked (yellow arrows), the NOPE negative carcinoma cells (blue arrow).

In situ analysis of NOPE mRNA expression was detected in a total of 32 patients (8.8%). It covered different forms of esophageal cancers, which is exemplary shown in Figs. 1 and 2. In Fig. 1, an EAC is depicted showing homogeneous NOPE mRNA expression in situ (small red dots) in the carcinoma cells (short arrows). These cells were surrounded by stromal cells such as fibroblasts and endothelia, which were NOPE mRNA negative (long arrow) (Fig. 1). In Fig. 2, a tubular-intestinal differentiated EAC is depicted which was heterogeneously positive for NOPE mRNA expression. To visualize the multiple wide lumen carcinoma glands, they were outlined in yellow. NOPE mRNA expressing cells were highlighted by yellow arrows and Nope negative carcinoma cells were highlighted by blue arrows (Fig. 2). NOPE expression was significantly more frequent in women (18.9%) than seen in men (6.9%), *P* = 0.032 (Table 1). A correlation between NOPE expression and additional clinical or histopathological data (age, pT stage, lymph node metastasis (pN)) was not detectable in cross table analysis.

### Correlation of NOPE with survival data

**Figure 3:**
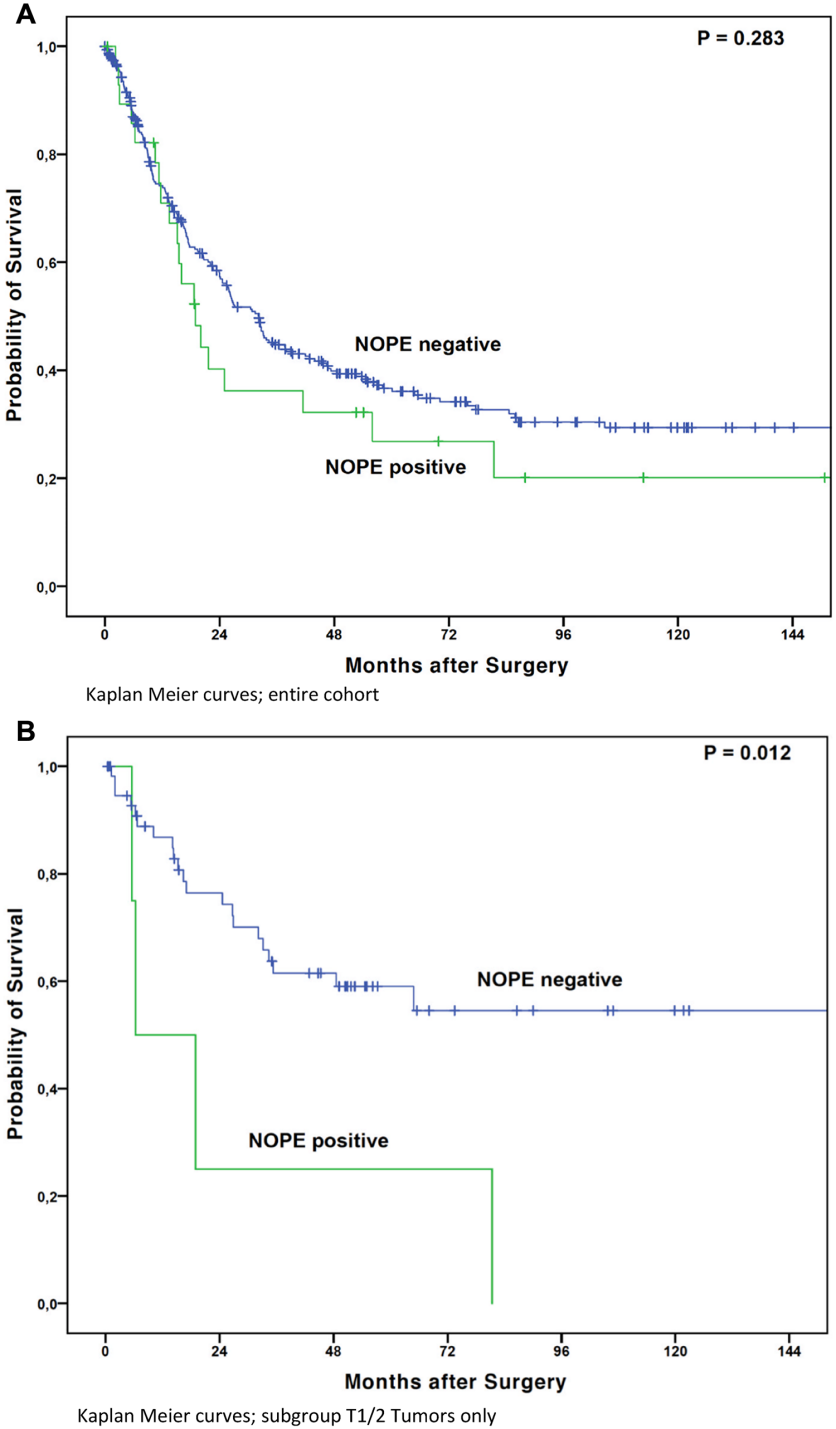

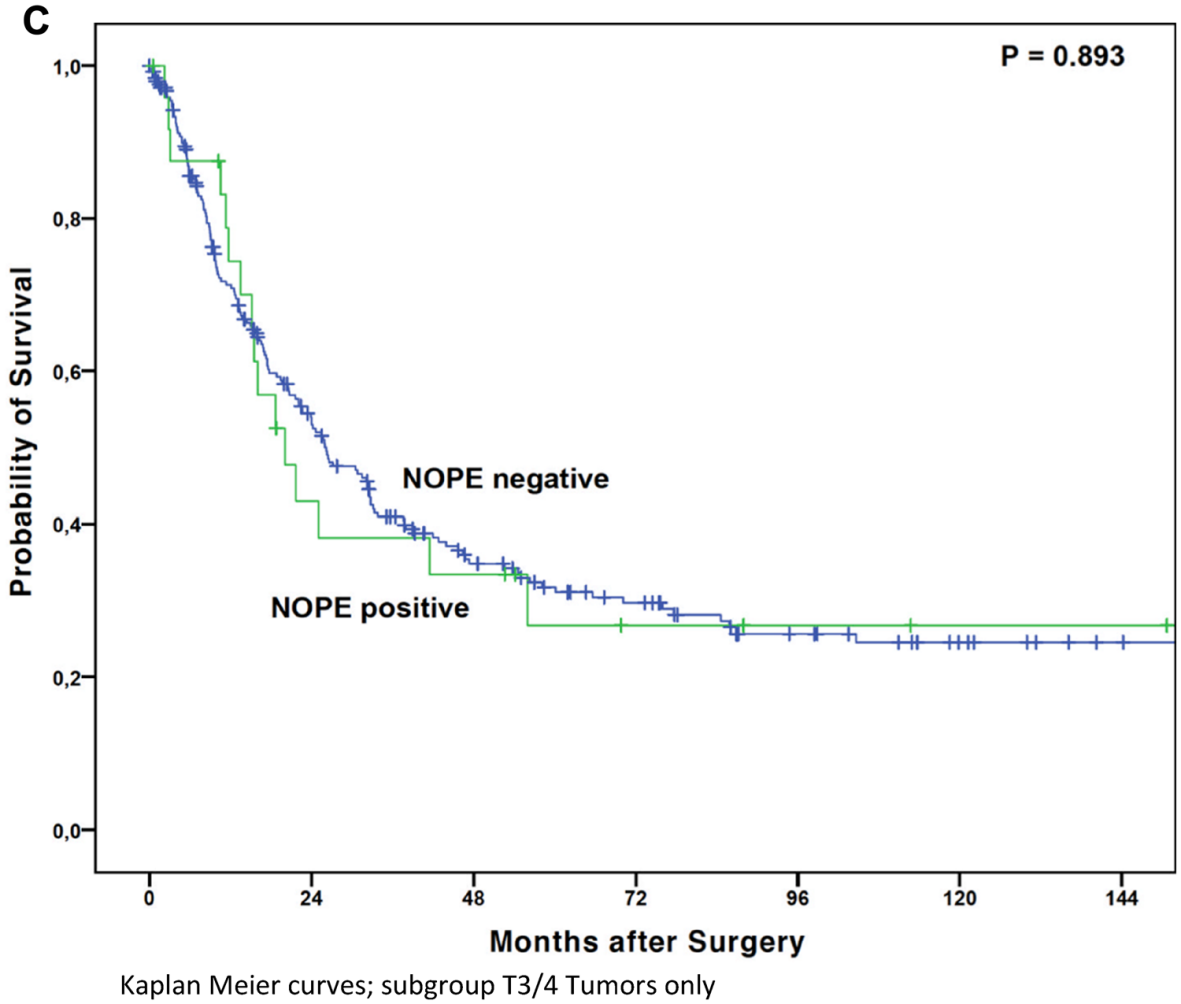
(**A**) Kaplan Meier curves; entire cohort. (**B**) Kaplan Meier curves; subgroup T1/2 Tumors only. (**C**) Kaplan Meier curves; subgroup T3/4 Tumors only.

The median overall survival (OS) in patients with NOPE positive tumors was numerically shorter than in those with NOPE negative tumors at 18.9 months (95% confidence interval (95% CI) 12.4–25.4 months) compared to 32.2 months (95% CI 26.7–37.6 months), respectively. This difference did not reach statistical significance, (*P* = 0.283) (Fig. 3A). However, considering only patients at early local tumor stages (pT1/2), NOPE expression was significantly associated with a shortened OS in this particular subgroup. Patients with pT1/2 tumors and presence of NOPE expression showed a median OS of only 6.3 months (95% CI 1.2–19.4 months) while the median OS was not reached in the NOPE-negative group (mean OS 117.1 months) (*P* = 0.012) (Fig. 3B). In advanced tumor stages, a survival difference between NOPE positive (median OS 20.1 months, 95% CI 11.7—28.4 months) and negative patients (median OS 26.0 months, 95% CI 19.6—32.3 months) could not be detected (*P* = 0.893) (Fig. 3C). There was a trend towards worse survival in lymph node negative (N0), patients in patients with NOPE positive tumors (median OS 21.7 months) vs. NOPE negative tumors (median OS 202.2 months) (*P* = 0.083). Neither administration of neoadjuvant treatment nor differences in sex affected the NOPE-dependent OS (data not shown). NOPE expression was not associated with the administration of neoadjuvant treatment (*P* = 0.134). Patients with NOPE positive tumors had a longer median survival in the T3/4 tumor category (median OS 20.1 months, 95% CI 11.7–28.4 months) than in the T1/2 group (median OS 6.3 months, 95% CI 0–19.4 months).

### Correlation of NOPE with other immunhistochemical markers

NOPE expression was significantly associated with the expression of IDO on tumor-infiltrating lymphocytes (TILs) (*P* = 0.013). IDO expression on TILs was seen in 59.6% of tumors and there was an expression on tumor cells in 9.2%. Additionally, we found an association of NOPE with ITGAV expression (detectable in 14.3% of all tumors) on tumor cells (*P* = 0.021).

## Discussion

In this first analysis of NOPE expression in EAC, we can show in a large patient cohort that the expression of NOPE has a negative prognostic impact and is associated with a detrimental prognosis in early invasive carcinomas.

Stage-specific treatment of esophageal carcinoma has yielded promising results in recent years^32^. However, there is conflicting data on the benefits of pre- or perioperative therapy in the small subset of patients with T2 tumors^33,34^. Here, we found that the presence of NOPE in precisely this early stage of the disease was associated with a very poor outcome. Due to a lower rate of lymph node metastasis and occult distant metastases, the T2 category usually has a prognostic advantage over T3/4 tumors and in an unselected cohort, the expected effect of neoadjuvant therapy is probably lower^14,15^. NOPE, if found in preoperative biopsy, could be a marker to seed out patients at risk for tumor progression and treat them with neoadjuvant therapy analogous to advanced stage patients, a collective in which positivity no longer appears to impact prognosis. Current guidelines on the treatment of esophageal carcinoma give no clear recommendation regarding perioperative treatment for T2 tumors^16,34^, stating lack of evidence as a reason. However, classifying patients into prognostic categories based on tumor stage alone may not do justice to the individual patient. To increase precision in pre-selecting patients at risk, numerous prognostic biomarkers have been evaluated in patients with esophageal carcinoma treated in curative intent^35^. A meta-analysis published in 2018 by Creemers et al. ^36^ identified a total of 82 unique biomarkers. The majority of the biomarkers examined in this analysis are involved in tumor cell proliferation, HER2, EGFR, cyclin D, KI67 and MTOR were among the most frequently reported in literature found by the authors. All these are factors involved in the development of invasive growth properties and metastasis^37^.

The functions of NOPE, although not yet entirely understood, likely lie in homotypic cell–cell contact and its similarity to neogenin and ‘deleted in colorectal cancer’ (DCC) suggest a role in axon guidance^38,39^, which in turn is a system associated with invasive growth properties and epithelial-mesenchymal transition (EMT)^40^. NOPE was first identified in the liver by microarray analysis of murine fetal livers^41^. It reappears when the liver faces severe damage such as acute or chronic cholestasis but diminishes after regeneration (unpublished data). Originally, we found NOPE to be expressed in murine depolarized adult hepatocytes after bile duct ligation^21^. Just recently, we have been able to demonstrate the role NOPE may play in the future as a novel marker in human HCC^23^. Similar to the clinically established tumor markers for HCC AFP and GPC-3, NOPE is highly expressed during fetal liver development but is undetectable in healthy adult hepatocytes. In mouse models, NOPE is specifically expressed in HCC and remains undetectable in normal liver and in precancerous lesions^22^. While we do not believe NOPE per se is the aggressor in these early stage esophageal carcinomas, it shows potential as a possible surrogate marker for impending loss of cell–cell contact, and appears to act as a harbinger of poor outcome in early stage EAC. The loss of epithelial apicobasal polarity, a system in which NOPE likely plays a critical role, is a key factor in the induction of oncogenesis and the development of the invasive capabilities of tumors that pave the way for metastasis. Wan et al. described how the basolateral cell polarity complex protein scribble, when present in the cytoplasm, stimulates a gene signature as well as a phenotype characteristic of epithelial to mesenchymal transition (EMT) and tumor cell invasiveness via AKT signaling^20^.

This is in line with our results of correlating NOPE expression with other markers in our cohort: We found a significant association with both ITGAV and IDO. ITGAV is a transmembrane glycoprotein responsible for cell-to-matrix binding, which is physiologically almost undetectable and has been found be a marker for tumor progression in multiple tumor entities^17^. It can be found in a number of advanced malignancies such as breast, pancreatic and colorectal cancer, typically being absent in early tumor stages^17^.

In our cohort, its expression was detectable in 14.3% of all tumors and was significantly associated with a shortened overall survival in patients that underwent primary surgery. This effect did not apply to the group of patients that received neoadjuvant treatment^17^. Interestingly, this is a similar finding to NOPE positivity in early stage tumors which themselves had also not received neoadjuvant treatment. Tumor cells with increased ITGAV expression show heightened cell migration in vitro and in vivo as well as an increased rate of cell proliferation^42^.

IDO is a known marker for poor prognosis in esophageal squamous cell cancer, whereas a positive effect has been shown for breast cancer^18^. In our cohort, IDO expression on TILs was seen in up to 59.6% of tumors (9.2% expression on tumor cells) and was, contrary to our findings regarding NOPE, associated with a positive outcome. This positive outcome was associated with IDO expression on TILs and was most pronounced in early stage tumors (pT1/2: 142.1 vs. 37.1 months, *P* < 0.001)^18^ again, in an area in which NOPE + tumors perform very poorly. The prognostic impact of IDO across all tumor stages regarding OS was not dependent on whether patients received neoadjuvant treatment or not and its expression remained a positive prognostic marker in both patient groups^18^. Overall, it appears that IDO, which is mainly found on inflammatory cells, when present, is unable to compensate for the pending negative course of the disease as indicated by NOPE positivity in tumor cells.

Limitations of our study are the retrospective study design and the fact we were unable to include patients who received neoadjuvant treatment and showed a complete tumor response as well as patients with advanced tumors that were ineligible for surgical therapy may have led to a selection bias. An additional limitation lies in the omission of a complete multivariate analysis, which we opted against due to some instances of missing pieces of data.

The NOPE- pT1/T2 cohort had an exceptionally good survival with 117.1 months, which is essentially in line with the survival benefit observed in early stage tumors showing IDO expression (ZITAT 15). The reasons for this very positive outcome remain unclear, however may be attributed in part to the center’s surgical expertise.

This is the first report of NOPE expression in a large group of patients in EAC and we are the first to describe a possible prognostic role for NOPE in a solid tumor. The relatively low frequency of NOPE expression was detectable due to the large sample size in this study and was associated with patient prognosis in pT1/2 tumors.

## Conclusions

Early stage (T1/T2), NOPE positive patients are at a high risk of tumor progression and show a worse outcome than patients with NOPE positive T3/T4 tumors. This patient group may benefit from neoadjuvant treatment analogous to advanced stage cancer. These findings warrant confirmation in a larger collective as this marker could contribute to advancing from stage-specific to individualized treatment approaches.
